# Evaluating the impact of a short bout of stair-climbing on creative thinking in a between-subjects pretest posttest comparison study

**DOI:** 10.1038/s41598-023-50282-2

**Published:** 2024-01-02

**Authors:** Chihiro Kawashima, Chong Chen, Kosuke Hagiwara, Tomohiro Mizumoto, Mino Watarai, Takaya Koga, Fumihiro Higuchi, Yuko Fujii, Emi Okabe, Shin Nakagawa

**Affiliations:** Division of Neuropsychiatry, Department of Neuroscience, Yamaguchi University Graduate School of Medicine, 1-1-1, Minami-Kogushi, Ube, Yamaguchi 755-8505 Japan

**Keywords:** Human behaviour, Cognitive neuroscience, Public health

## Abstract

Recent studies have indicated potential links between short bouts of physical activity like stair-climbing and enhanced creative thinking. However, previous research featured limitations, such as using an uncommon 3 flights round-trip design and lacking baseline creative thinking evaluations. To rectify these limitations and build a more comprehensive understanding, the present study adopts a between-subjects pretest posttest comparison design to scrutinize the effects of ascending stair-climbing on both divergent and convergent thinking. 52 subjects underwent a pretest, followed by random assignment to one of four interventions: ascending stair-climbing for 2, 5, or 8 flights, or taking an elevator for 8 flights, before progressing to a posttest. The results revealed a notable improvement in convergent thinking, measured by the increased number of solved matchstick arithmetic problems (d = 1.165), for participants who climbed 2 flights of stairs compared to those who took the elevator. However, climbing 5 or 8 flights showed no such impact on convergent thinking, and stair-climbing, regardless of the number of flights, did not influence divergent thinking. These findings underscore the utility of brief stair-climbing as an accessible means to enhance convergent thinking in everyday settings, providing a nuanced insight into the relationship between physical activity and creative thinking processes.

## Introduction

Divergent and convergent thinking stand as pivotal components of creative thinking^1–3^. Divergent thinking involves the generation of diverse, novel solutions, facilitated by cognitive flexibility and associative abilities^4^. Conversely, convergent thinking aims to discern a singular resolution, bolstered by higher-order problem-solving skills integral to fluid intelligence^3,5^. Contemporary research underscores the potential of brief sessions of aerobic exercises like walking, cycling, and aerobic dance to enhance creative cognition, thus presenting a viable strategy for bolstering creative thinking in everyday contexts^6–8^. A study in a real-world context revealed that, compared to elevator use, descending and ascending three flights of stairs amplified divergent thinking, demonstrated by elevated originality scores on the Alternate Uses Test (AUT^9^), while leaving convergent thinking, evaluated through matchstick arithmetic problems, unaffected^10^.

However, the aforementioned study utilized a within-subjects crossover posttest design and did not assess creative thinking at baseline. Although a crossover pretest posttest comparison design yields more conclusive evidence, it necessitates that each participant undergoes evaluation four times, engendering not only learning and practice effects but also imposing an increased burden on the subjects^11^. Consequently, the researchers opted for a within-subjects crossover posttest comparison design in the prior study and administered creative thinking tests solely post-intervention, i.e., after stair-climbing or elevator use. Thus, it remains ambiguous whether the enhanced divergent thinking observed is attributable to the positive effects of stair-climbing or the potential inhibitory effects of elevator use, involving confinement within a limited space. Moreover, to mirror the intensity of another study where four minutes of walking boosted divergent thinking^12^, stair-climbing was structured as descending and then ascending three flights. However, round-trip stair-climbing is seldom practiced in real life. These constraints necessitate further meticulously structured studies to re-examine the impact of stair-climbing on creative thinking.

In this context, the present study aimed to address these limitations and explore the impact of ascending stair-climbing on creative thinking, employing a between-subjects pretest posttest design. Implementing a refined between-subjects pretest posttest design, the study is designed to precisely delineate the enhancements in divergent and convergent thinking that could be uniquely attributed to ascending stair-climbing. The emphasis was placed on ascending stair-climbing, primarily due to its more strenuous nature compared to descending and the logistical convenience it offered, allowing subjects to return promptly to our ninth-floor laboratory for the post-intervention creative thinking assessments.

Our hypothesis asserts that ascending stair-climbing holds the potential to significantly enhance both divergent and convergent thinking. This assertion is substantiated by three strands of neurobiological evidence. First, activities requiring meticulous control, like stepping over obstacles, have been linked to increased activation of the prefrontal cortex (PFC^13,14^), a key brain area involved in divergent thinking^15^ and problem solving^16^. Second, the heightened functional connectivity between the hippocampus and cortical regions, observed during low-intensity activities, is likely to contribute to improvements in memory processes essential for creative cognition^17^. Third, dopamine release in the PFC as well as the striatum during physical activity^18,19^ is believed to augment cognitive flexibility^20–22^, components crucial for creative thinking. This synthesis of neurobiological insights elevates the premise that ascending stair-climbing may serve as an innovative catalyst for enhancing creative cognitive faculties, highlighting its potential as a pivotal strategy in the enrichment of creative thought processes.

## Methods

### Subjects

Based on a priori power analysis, a sample size of 48 subjects is deemed necessary to detect a medium effect size interaction between within-subjects and between-subjects factors, utilizing repeated measures ANOVA (f = 0.25), across 2 time points and 4 interventions, with a power of 0.8 and a significance level of 0.05, 2-sided. To accommodate potential dropouts or data discrepancies, we enrolled 52 subjects, assigning 13 to each intervention.

Eligibility was limited to individuals aged 20–29 years. Those currently experiencing psychiatric disorders, intending to undergo psychiatric assessment, having participated in our preceding study, or being employed by our department were excluded. Of the initial 52 subjects, one, assigned to 8-flight stair-climbing, was omitted from the analysis due to procedural inconsistencies (details below), resulting in a total of 51 subjects (26 males, 25 females, age 21.84 ± 1.83 years). The study was approved by Yamaguchi University Hospital Institutional Review Board and conducted following the Declaration of Helsinki. Subjects gave written informed consent before the study.

### Procedure and interventions

Subjects were instructed to ensure ample sleep and abstain from intensive physical activities, smoking, and caffeine consumption for a minimum of two hours preceding the study. Upon arrival and receiving detailed explanations of the study, subjects provided written informed consent and responded to demographic queries and compliance with preparatory instructions. These procedures collectively consumed approximately 15–20 min.

**Figure 1:**
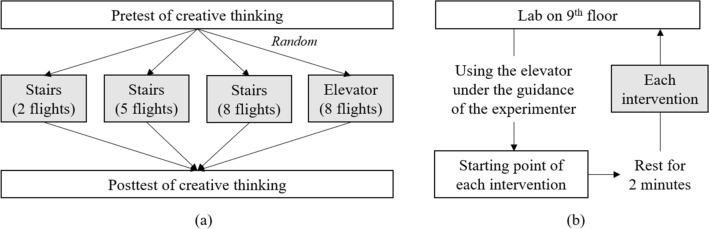
The procedure of the study. (**a**) Study design and randomization. (**b**) Procedure of the intervention.

We employed a between-subjects pretest posttest comparison design (Fig. 1a). Initially, participants completed a creative thinking pretest, subsequently being randomized to one of four interventions: stair-climbing for 2, 5, or 8 flights, or taking the elevator for 8 flights, with 13 subjects in each group. The decision for 2-flight stair-climbing was informed by our pilot survey, wherein the prevalent response to “Until what floor do you use the stairs?” was “third floor” (i.e., 2 flights). The 5-flight stair-climbing was conceptualized to mirror the intensity of the 3-flight round-trip stair-climbing from Matsumoto et al.^10^. Given estimations that ascending stair-climbing bears approximately 1.8 times the intensity of descending^23^, a 3-flight round-trip equates to roughly 4.7 flights of ascending; hence, we rounded up to 5 flights. To explore the impact of climbing more than 5 flights, we settled on 8 flights for its symmetry to 2 flights in relation to 5 flights and due to the habitual stair use by several ninth-floor employees in our research building. As a control intervention, ascending 8 flights via elevator was selected.

For the intervention procedure, following the pretest of creative thinking that lasted 4 min, subjects assessed their immediate mood regarding pleasure, relaxation, and vigor with a visual analog scale^6,24,25^ and donned an Apple Watch (Apple Inc.) to monitor duration and heart rate in our ninth-floor laboratory. The mood test generally took less than 30 s. Subsequently, they received their randomization assignments and were escorted by the experimenter via elevator to their respective intervention starting points (e.g., seventh floor for 2 flights stair-climbing, Fig. 1b). A 2-min rest at the starting point mitigated the impact of any preceding physical activity. Maintaining a natural pace, participants commenced their interventions immediately after their rest. Participants’ mood was reassessed post-intervention, followed by the posttest of creative thinking.

However, preliminary trials revealed a brief period of breathlessness and impaired concentration following 8-flight stair-climbing. A 2-min recovery period was instituted for these participants before conducting the posttest. Due to procedural error, one participant from this group who didn’t receive the recovery period was excluded from the data analysis, reducing the group size to 12.

Each session (pretest, intervention, posttest), the overall duration of which ranged from approximately 15–25 min, was conducted twice, assessing divergent then convergent thinking, with divergent thinking evaluated first to prevent any potential impact on motivation and mood from the more challenging convergent thinking tasks^12^. A consistent intervention was maintained for both thinking evaluations with a 15-min washout period in between.

The intervention occurred between 9:00–11:00 am and 13:00–17:00 pm within a medical research building, a period during which traffic was minimal as medical staff are predominantly engaged with outpatient and inpatient wards during the day.

### Divergent thinking

We utilized the AUT to evaluate divergent thinking. Participants were instructed to brainstorm as many unconventional, inventive uses for three commonplace items (e.g., "newspaper") as they could within a four-minute frame, documenting their responses on a blank A4-sized paper. Two distinctive object sets were utilized for the pretest and posttest, with the order being randomized. The specific instructions used were as follows:

“In this test, please write down as many unusual, creative, and unique uses as you can think of for the everyday items that will be presented to you shortly. An example question and its answer are as follows. You will be given three everyday items, so please provide your responses for each one. You have a total of 4 minutes for this task.”

As the example question, “button” was used and the example answers presented were, a doorknob for a toy house; eyes for a doll; a small sieve; a musical instrument; a game piece (e.g., a checker); a peephole for a front door; a marker dropped along a path.

Consistent with previous studies^6,10,26^, fluency was defined as the quantity of unusual uses conceived for the three items. Common uses of each item and duplicate responses were excluded from this count. Flexibility was characterized by the number of conceptual categories that resulted into functional distinctions into which the generated uses could be sorted into; for example, “wiping a window” and “cleaning the wall” would fall into the same conceptual category. Originality was assessed by tallying the number of distinct conceptual categories identified. Categories shared by multiple participants were excluded from the count. An original use was counted for a participant only if it was unique to that individual, not if it was generated by multiple participants.

The individual responsible for coding AUT was trained using data from a preceding research^6^. Post-training, the coder achieved near or full concurrence with the prior study’s coder, as evidenced by Cohen’s κ^27^, with κ = 1 for fluency, 0.828 for flexibility, and 0.821 for originality. The coding was performed while the coder remained unaware of the participants’ randomization assignments.

### Convergent thinking

To assess convergent thinking, we utilized the matchstick arithmetic insight problems^28^. Subjects were provided with incorrect formulas in Roman numerals constructed with matchsticks and were tasked with rectifying the formulas by adjusting the position of a single stick. For instance, for VII = II + III, subjects would modify “VII” to “II” to correct the equation. In alignment with^10^, three problems were selected, and the resolution time was capped at four minutes. Subjects underwent training to ensure proficiency in reading Roman numerals before the convergent thinking pretest. The quantity of accurate solutions was incorporated into the data analysis. The specific instructions used for this test were as follows:

“Move only one matchstick to make the equation correct. The total time limit is 4 minutes. If you cannot solve the problem, you may proceed to the next question; furthermore, within the time limit, you can attempt the unsolved question as many times as you like. Please do not give up along the way and do your best until the end”.

### Statistical analysis

Analyses were performed utilizing IBM SPSS Statistics 26.0. To discern the characteristic and intensity of each intervention, initial comparisons of heart rate (HR) and time duration, and mood alterations from pre- to post-intervention were executed using one-way ANOVA and repeated measures ANOVA, respectively. The average of HR, time duration, and mood alterations for both divergent and convergent thinking sessions were considered for analysis as the interventions were consistent across sessions. Subsequently, between-group differences in alterations of creative thinking scores from pre- to post-intervention were compared using repeated measures ANOVA. Upon identifying a significant time*intervention interaction, alterations in creative thinking scores for each stair-climbing group were contrasted against the elevator group utilizing t-tests and Wilcoxon signed-rank tests, where data demonstrated non-normal distribution. Effect sizes were determined using GPower Version 3.1.9.7^29^ and a significance level of 0.05 was used.

The inclusion of three stair-climbing levels facilitated the exploration of potential dose-dependent relationships (linear, U-shaped, or inverse U-shaped) between stair-climbing levels and alterations in creative thinking scores. To examine such relationships, data were fitted to linear and quadratic regression models, treating the control intervention as zero flights.

In addition, to investigate the influence of exercise intensity and mood on the creative thinking effects of stair-climbing, we conducted correlation analyses between heart rate/mood changes and changes in creative thinking measures. We used either Pearson or Spearman correlation, depending on data normality. Variables that exhibited significant correlations with changes in creative thinking were then included as covariates in a two-way repeated measures ANOVA to confirm their role in accounting for the stair-climbing-induced creative thinking enhancements. To explore the extent of covariation among the three measures of divergent thinking, we also conducted additional correlation analyses for these measures.

## Results

### Characteristics of each intervention

**Figure 2:**
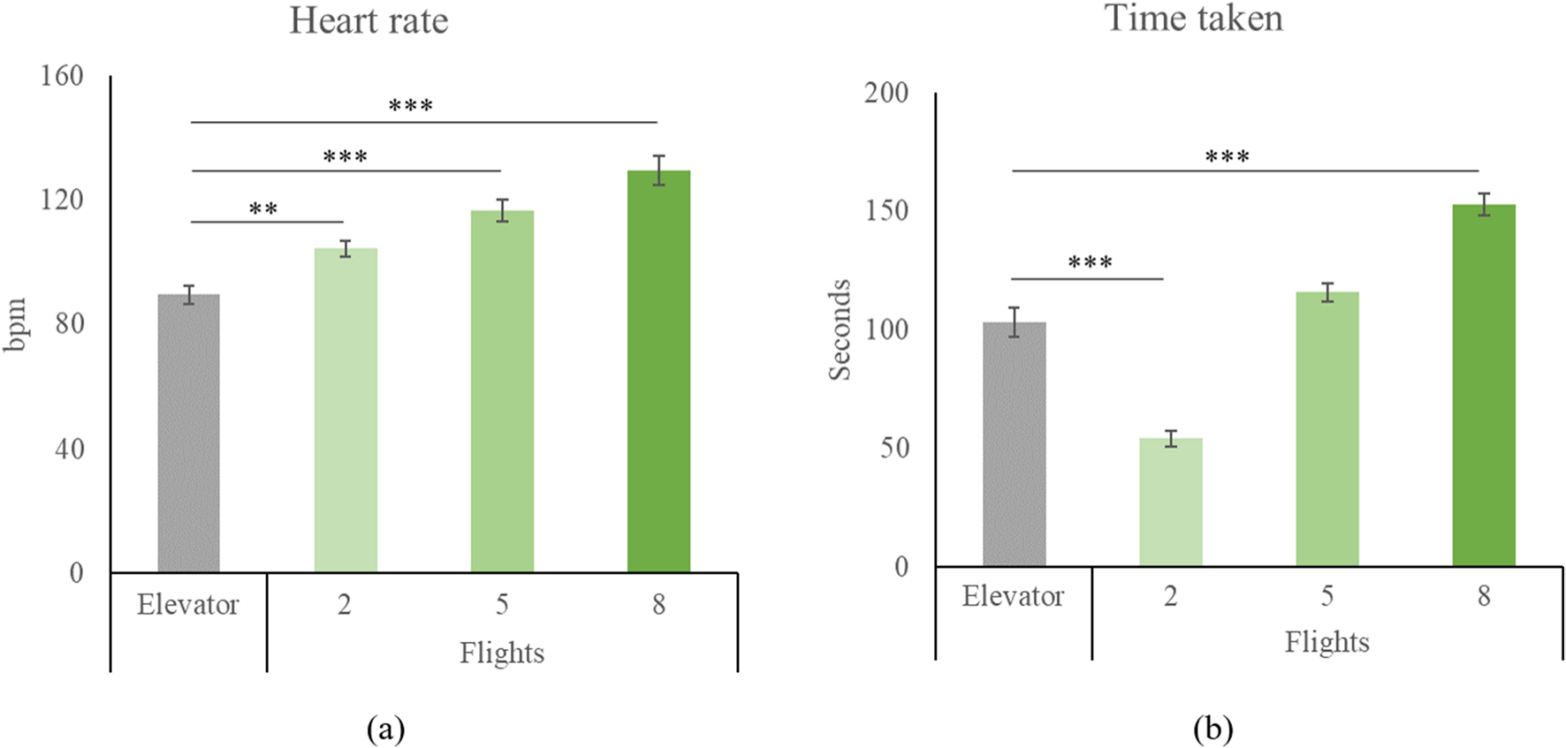
Heart rate and time taken for each intervention. Data shown as mean ± SE. ** *p* < 0.01, *** *p* < 0.001.

The statistical results are summarized in Table 1. The results of HR and time duration for each intervention are shown in Fig. 2. A one-way ANOVA revealed a significant between-group difference for both HR (F = 24.113, *p* < 0.001, f = 1.240) and time take (F = 75.959, *p* < 0.001, f = 2.194). Compared to the elevator group, all stair-climbing groups exhibited elevated HR (all *p* < 0.001, d = 1.494, 2.347, and 2.949 for 2, 5, and 8 flights, respectively). Climbing 2 flights was quicker (*p* < 0.001, d = − 2.701) while stair-climbing for 8 flights was more time-consuming (*p* < 0.001, d = 2.583) in comparison to elevator usage.

**Table 1:** Statistical results.

		One-way ANOVA or two-way repeated measures ANOVA: Time*intervention interaction	Compared to elevator use		
			Climbing 2 flights	Climbing 5 flights	Climbing 8 flights
Heart rate		F = 24.113, *p* < 0.001, f = 1.240	*p* < 0.001, d = 1.494	*p* < 0.001, d = 2.347	*p* < 0.001, d = 2.949
Time taken		F = 75.959, *p* < 0.001, f = 2.194	*p* < 0.001, d = − 2.701	*p* = 0.091, d = 0.691	*p* < 0.001, d = 2.583
Mood	Pleasure	F = 4.224, *p* = 0.010, f = 0.519	*p* = 0.234, d = 0.479	*p* = 0.407, d = 0.331	*p* = 0.042, d = -0.862
	Relaxation	F = 0.836, *p* = 0.481, f = 0.232	–	–	–
	Vigor	F = 1.179, *p* = 0.328, f = 0.274	–	–	–
Divergent thinking	Fluency	F = 0.271, *p* = 0.846, f = 0.132	–	–	–
	Flexibility	F = 0.252, *p* = 0.859, f = 0.128	–	–	–
	Originality	F = 2.616, *p* = 0.062, f = 0.408	–	–	–
Convergent thinking		F = 3.170, *p* = 0.033, f = 0.445	*p* = 0.003, d = 1.165	*p* = 0.101, d = 0.686	*p* = 0.225, d = 0.579

**Figure 3:**
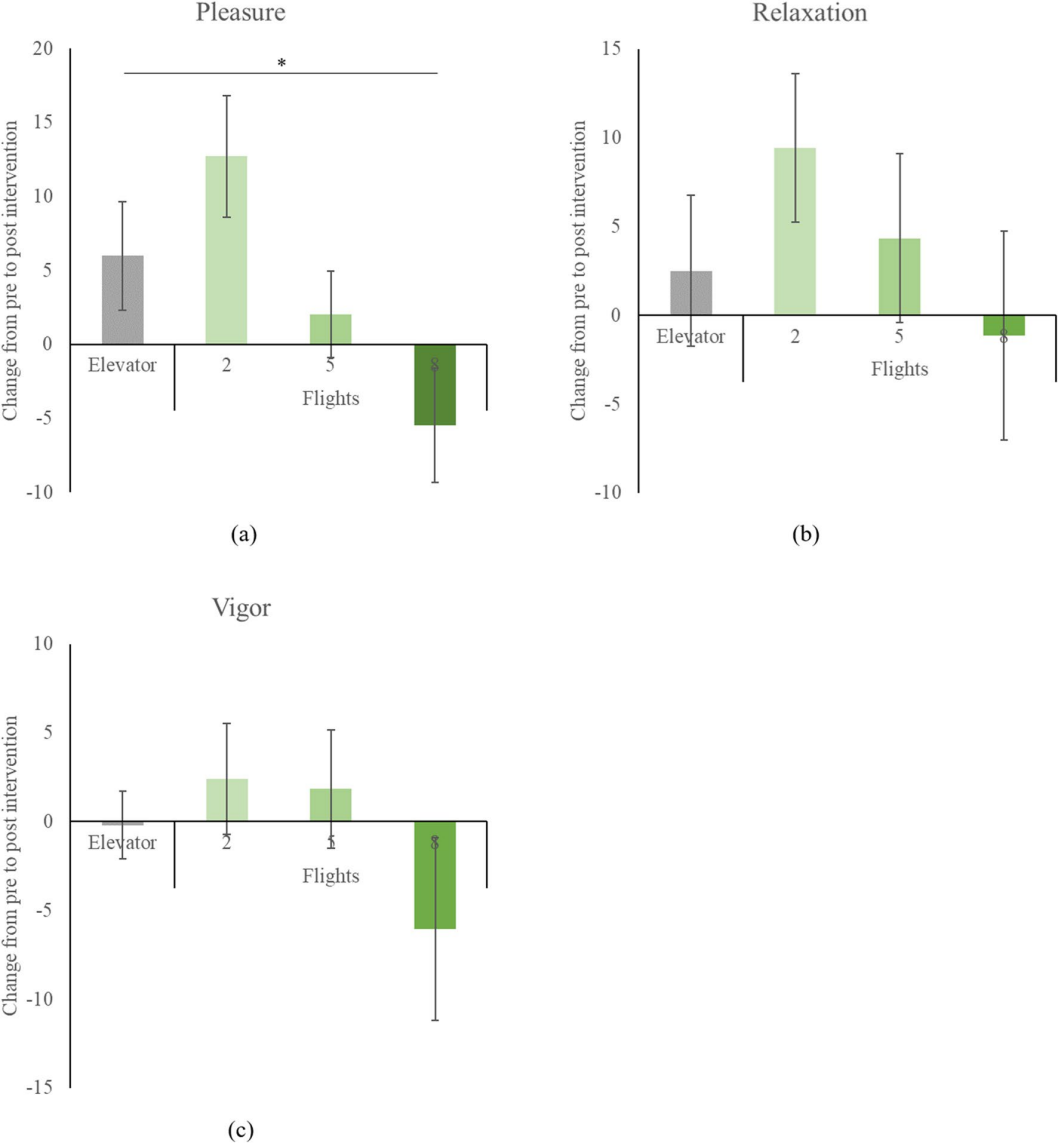
Mood changes from pre- to post-intervention for each intervention group. Data shown as mean ± SE. *p < 0.05.

Alterations in mood from pre- to post-intervention are illustrated in Fig. 3. Two-way repeated measures ANOVAs disclosed a significant time*intervention interaction for pleasure (F = 4.224, *p* = 0.010, f = 0.519), albeit not for relaxation (F = 0.836, *p* = 0.481, f = 0.232) or vigor (F = 1.179, *p* = 0.328, f = 0.274). In comparison to the elevator group, climbing 8 flights led to a decrease in pleasure (t = − 2.153, *p* = 0.042, d = -0.862).

### Creative thinking

**Figure 4:**
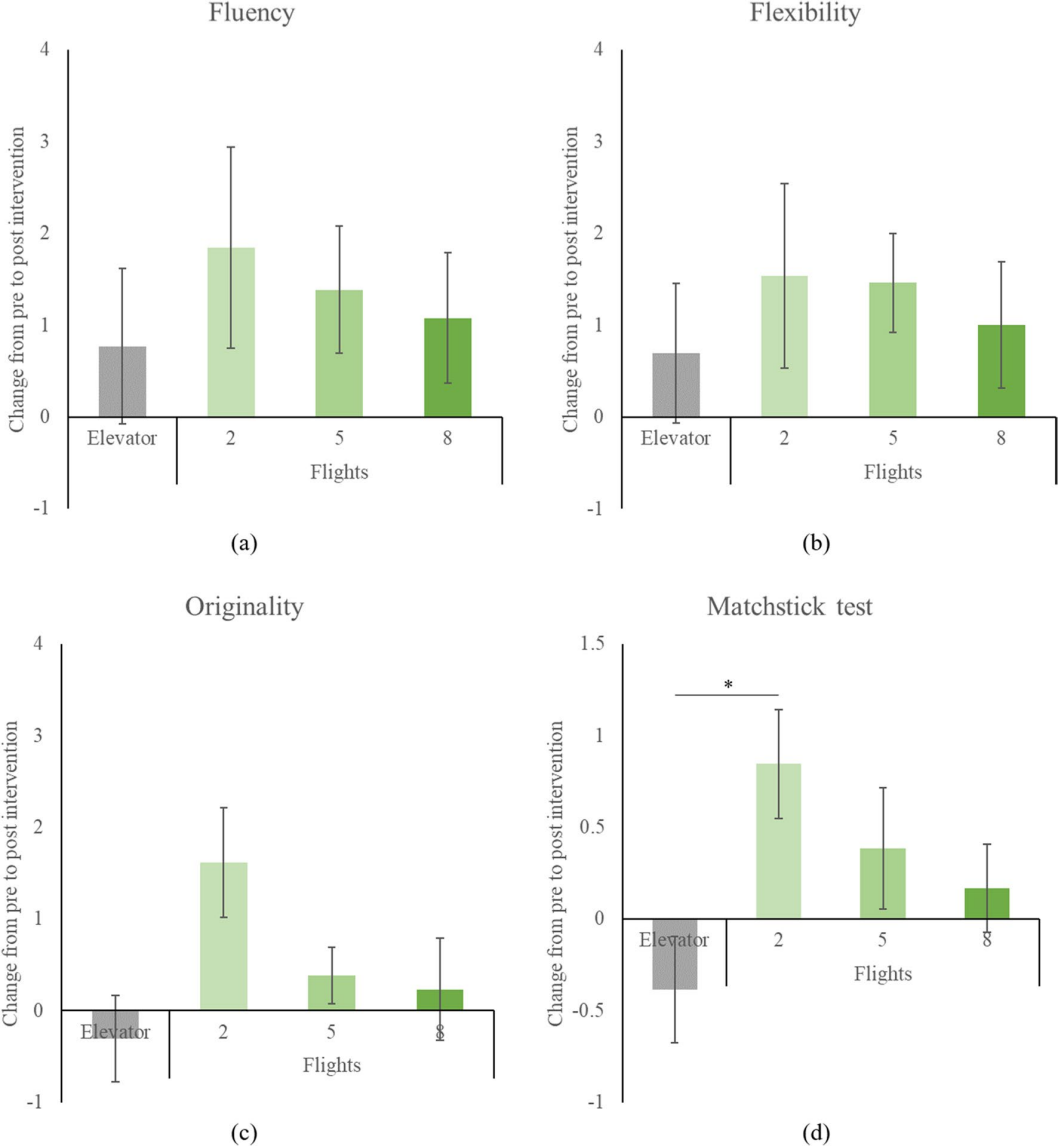
Changes of creative thinking from pre- to post-intervention for each intervention. (**a**–**c**), AUT. (**d**), Matchstick test. Data shown as mean ± SE. * *p* < 0.05.

Changes in the scores of creative thinking from pre- to post-intervention are shown in Fig. 4. Two-way repeated measures ANOVAs revealed no significant time*intervention interaction for either fluency (F = 0.271, *p* = 0.846, f = 0.132) or flexibility (F = 0.252, *p* = 0.859, f = 0.128). Nevertheless, the time*intervention interaction approached a trend towards significance concerning originality (F = 2.616, *p* = 0.062, f = 0.408).

Regarding convergent thinking, two-way repeated measures ANOVA indicated a significant time*intervention interaction for the matchstick test score (F = 3.170, *p* = 0.033, f = 0.445). Compared to taking the elevator, stair-climbing for 2 flights led to an elevation in the matchstick test scores (Mann–Whitney U = 29.0, *p* = 0.003, d = 1.165), whereas stair-climbing for 5 (U = 52.0, *p* = 0.101, d = 0.686) or 8 flights (U = 55.0, *p* = 0.225, d = 0.579) did not exhibit a significant increase.

Subsequent curve fitting corroborated the aforementioned results. In terms of changes in the scores of divergent thinking, neither linear nor quadratic curves showed significance for fluency, flexibility, or originality (all *p* > 0.14). Conversely, an inverse U-shaped quadratic relationship was discerned for alterations in the convergent thinking score (R-square = 0.126, *p* = 0.036).

Importantly, there were no pre-intervention differences in the scores of either divergent or convergent thinking (Figure S1).

### Association between heart rate/mood change and changes in creative thinking

**Figure 5:**
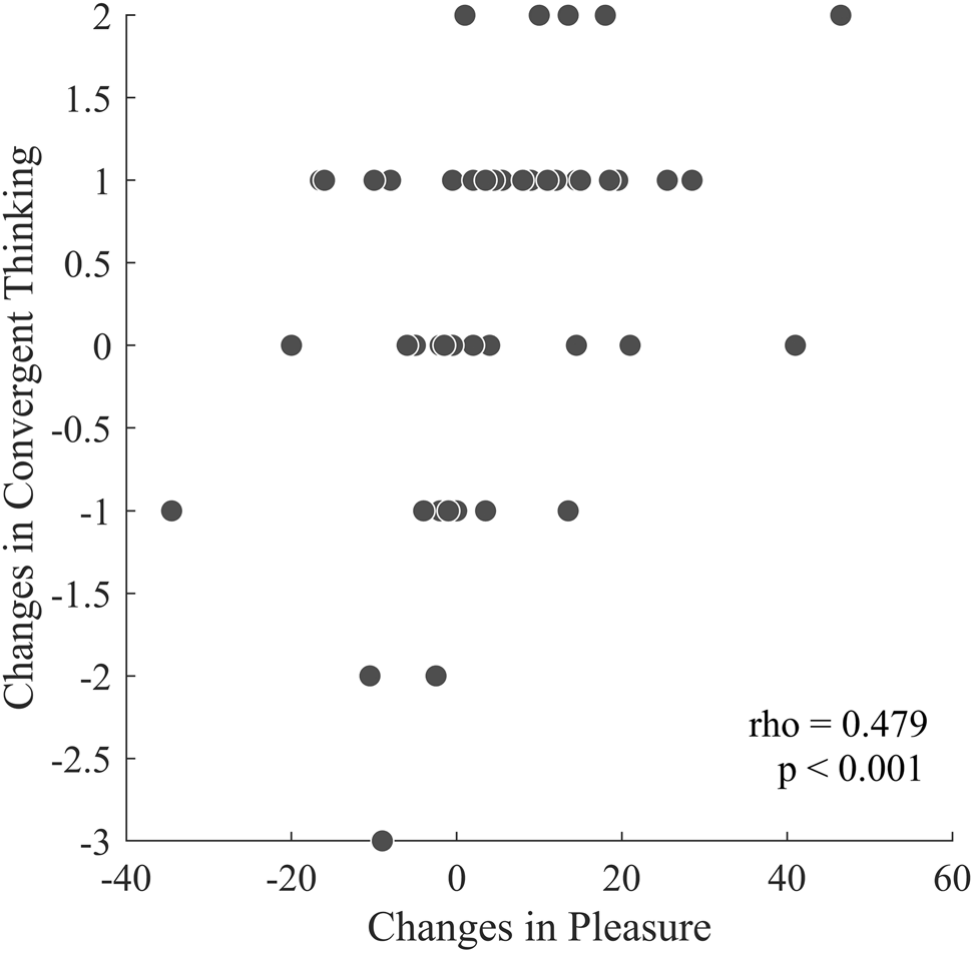
Scatterplot of the correlation between changes in feelings of pleasure and changes in convergent thinking (matchstick test).

Table 2 presents the results of correlation analyses between mood/heart rate and divergent/convergent thinking measures. In summary, heart rate did not show significant correlations with any divergent or convergent thinking measures. The only notable correlation was between changes in feelings of pleasure and improvements in convergent thinking (rho = 0.479, *p* < 0.001), suggesting that enhanced pleasure was associated with better convergent thinking. The scatterplot of this relationship is provided in Fig. 5.

**Table 2:** Correlation between heart rate/mood and creative thinking (n = 51).

		Heart rate	Mood changes			Changes in divergent thinking			Changes in convergent thinking (Matchstick test)
			Pleasure	Relaxation	Vigor	Fluency	Flexibility	Originality	
Heart rate		1	− 0.388**	− 0.019	− 0.134	0.146	0.122	0.096	0.126
Mood changes	Pleasure		1	0.120	0.248	0.044	0.064	0.105	0.479***
	Relaxation			1	0.533***	0.127	0.051	− 0.037	0.213
	Vigor				1	− 0.060	− 0.157	− 0.105	0.221
Changes in Diverged thinking	Fluency					1	0.952***	0.250	0.152
	Flexibility						1	0.322*	0.215
	Originality							1	0.169
Changes in convergent thinking (Matchstick test)									1

*p < 0.05, **p < 0.01, ***p < 0.001.

Since the most significant changes in pleasure and matchstick scores occurred after climbing 2 flights of stairs, we hypothesized that changes in pleasure might explain the improved convergent thinking after climbing 2 flights. To test this, we included changes in pleasure as a covariate in a two-way repeated measures ANOVA involving only the elevator and two flights groups.

Before adding the covariate, the time*intervention interaction was significant (F = 8.828, *p* = 0.007, f = 0.607). After incorporating changes in pleasure as a covariate, the interaction remained significant (F = 6.738, *p* = 0.016, f = 0.542), indicating that changes in pleasure did not eliminate the effect of climbing 2 flights of stairs on matchstick test scores. However, it did reduce the effect size by approximately 11%.

### Correlation among the three measures of AUT

As reported in Table 2, changes in fluency was significantly associated with changes in flexibility (r = 0.952, *p* < 0.001), while changes in flexibility was significantly correlated with changes in originality (r = 0.322, *p* = 0.021).

## Discussion

In the present study, we investigated the potential cognitive benefits of different intensities of stair-climbing, ranging from 2 to 8 flights. One key observation was the enhancement of convergent thinking following a 2-flight stair climb, as evidenced by improved matchstick test scores (d = 1.165). However, this boost was not observed for longer stair climbs, nor was there any influence on divergent thinking across all stair-climbing intensities.

Our findings of enhanced convergent thinking through brief stair-climbing contrast starkly with the previous study employing a 3-flight round-trip stair-climbing design^10^. The prior study indicated a potential enhancement in divergent thinking, which wasn't replicated in the present study. We hypothesized that climbing five flights of stairs would mimic the intensity experienced during three flights of round-trip stair-climbing and introduced two more levels of stair-climbing, i.e., two and eight flights, to explore any potential dose-dependent effects. Contrary to our expectations, divergent thinking remained unaffected at all tested intensities of stair-climbing. This raises the speculation that the elevation in divergent thinking observed in the study by Matsumoto et al.^10^ may be partly confounded by an inhibitory impact induced by elevator use. That is, the confines of an elevator space may have played a role in impacting the cognitive processes associated with divergent thinking. Additionally, the very act of using an elevator, typically associated with efficiency and routine, might contribute to a mindset focused on swiftly moving from one point to another. Such a utilitarian perspective may inadvertently stifle the open and relaxed mental state often conducive to divergent thinking. It is important to note that, as depicted in Fig. 2, the use of elevator in the current study did not result in a deterioration of mood.

Utilizing the age-predicted maximal HR formula (HR_max_ = 208−(0.7 × age)^30^), we observed the mean HR during stair-climbing for 2, 5, and 8 flights in our study to be 54.2%, 60.7%, and 67.7% HR_max_, respectively. According to the exercise intensity classification method used by the American College of Sports Medicine^31^, these values correspond to intensities of very light, light, and moderate, respectively. The enhancement of originality, deemed a pivotal aspect of divergent thinking, has been documented to occur during 4 min of walking at a natural pace—a typically very light intensity activity^12^. However, this enhancement is seemingly absent during engagement in physical activities of higher intensities: examples include 15 min of treadmill walking at 60.0% HR_max_ (light in intensity^32^), 44 min of treadmill walking at 60–70% HR reserve (vigorous in intensity^33^), 6 min of maximal effort cycling^34^, and a 15-min graded exercise test^6^. Interestingly, a different form of activity, 15 min of free dance with music, which is more enjoyable and less constrained, has been reported to improve originality^7,8^. In conjunction with our findings—which reveal a trend towards improved originality in less than one minute of two-flight stair-climbing at very light intensity—these reports prompt contemplation over the potentially superior efficacy of prolonged physical activity at very light intensity and enjoying, unrestricted physical activities for enhancing divergent thinking. This highlights a critical avenue for further exploration, potentially yielding insights beneficial for strategic incorporation of physical activity for cognitive enhancements in divergent thinking.

The exclusive enhancement of convergent thinking by stair-climbing for 2 flights, measured by the significant improvement in the matchstick test (d = 1.165), provides a novel perspective. This peculiarity also raises queries about the optimal intensity of physical exertion conducive to cognitive augmentation, especially when the elevation of convergent thinking is not apparent post more intensive stair-climbing (5 and 8 fights). An intriguing dimension is added by the identification of an inverse U-shaped quadratic relationship, suggesting that there might exist an optimal level of physical exertion which is task-dependent and exceeding it may not result in further cognitive enhancements or may even be detrimental^35^.

Previous studies have reported that convergent thinking remains unaffected by 15 min of treadmill walking at 60.0% HR_max_ (classified as light in intensity^32^) and by a 15-min graded exercise test^6^. Similarly, convergent thinking showed no significant change in Matsumoto et al.^10^ involving round-trip stair-climbing, which generated 56.8% HR_max_, positioning it near the threshold between very light and light intensities.

However, contrasting these reports, our current study yielded evidence of enhanced convergent thinking following stair-climbing for 2 flights, compared to taking the elevator. This new finding prompts speculation that engaging in physical activity at very light intensities may offer more effective enhancements to convergent thinking. This hypothesis seemingly contradicts the findings of Oppezzo and Schwartz^12^, who observed no impact on convergent thinking following four minutes of walking at a natural pace. However, it is crucial to note that in the study by Oppezzo and Schwartz^12^, assessments of convergent thinking were conducted during walking. The discrepancy between their findings and ours may thus be attributed to the known variance in cognitive function effects observed during versus post exercise^36^. This variance indicates a nuanced relationship between exercise intensity, timing, and cognitive enhancements, necessitating further investigations to elucidate the optimal conditions promoting convergent thinking through physical activity.

Another result worth mentioning is that both divergent and convergent thinking scores trended similarly, particularly noting the changes in originality after climbing two flights of stairs. The inclusion of multiple stair-climbing groups in our study may have limited our ability to detect a significant effect specifically attributable to climbing two flights. Notably, a direct comparison between the originality score changes in the elevator group and the two-flight group reveals a statistically significant effect (*p* = 0.026). This finding suggests that future research should focus specifically on investigating the potential of two-flight stair climbing to enhance divergent thinking. An additional consideration when interpreting our results is the serial order effect in divergent thinking. This effect arises when participants are asked to generate as many uses as possible for an object without specific guidelines on the nature of these responses. Initially, more common and conventional responses emerge, which are typically less original. As these common ideas are exhausted, participants are compelled to apply greater cognitive effort, leading to increasingly original responses over time. This progression often results in a decrease in fluency and flexibility^37–39^. However, in our study, we specifically instructed participants to think of “as many unusual, creative, and unique uses” as possible, and we excluded common uses from our analysis. Consequently, the serial order effect is less likely to be a significant factor. Given these modifications, the observed lack of impact on fluency and flexibility, coupled with the trend in originality, are consistent with existing literature and our research methodology. This suggests that specific instructions focusing on originality, along with factors such as test duration and the serial order effect, could partly account for our findings.

While it has long been posited that a positive mood is a catalyst for creative thinking^40,41^, interventions such as aerobic dance^42^ and graded exercise tests^6^ that ostensibly enhance mood, do not seemingly influence the originality inherent in divergent or convergent thinking. However, an exploratory analysis in one of these studies did find a correlation between mood changes and convergent thinking performance, indicating that greater mood improvement was associated with better convergent thinking^6^. Similarly, we observed a significant correlation between changes in feelings of pleasure and changes in convergent thinking. Nonetheless, when we included mood changes as a covariate in our two-way repeated measures ANOVA, the convergent-thinking enhancement effect of stair-climbing for 2 flights remained significant, albeit with a slightly reduced effect size of approximately 11%. These findings suggest that the enhancement of convergent thinking through stair-climbing for 2 flights is largely independent of mood changes. This presents a paradoxical scenario, the mechanistic underpinnings of which necessitate exploration in future research endeavors. Additionally, our current research indicates that stair-climbing for 8 flights diminishes feelings of pleasure, but does not detrimentally affect either divergent or convergent thinking. This suggests the possibility that the adverse effects of a decline in mood on creative thinking might be mitigated or neutralized by the concurrent positive influences of physical activity. This interplay between mood, physical activity, and cognitive functionality warrants a deeper exploration to unravel the nuanced dynamics governing these variables.

Another factor that can influence the cognitive impact of acute exercise is the concept of recovery, which refers to the time that has passed since the termination of the exercise session^43–45^. Consistent with our previous study^10^, we administered the creative thinking tests immediately after the interventions (i.e., for 2 and 5 flights) and a brief mood test that typically takes less than 30 s. Although it is assumed that the cognitive effects of exercise at low intensities and shorter durations may decline rapidly, it remains a subject for future research to investigate the duration of the convergent thinking-enhancing effect of climbing 2 flights. Conversely, for the 8-flight stair-climbing intervention, we introduced a 2-min recovery period before conducting the creative thinking tests. This decision was based on feedback from our pilot experiment, where participants reported breathlessness and impaired concentration immediately after completing the 8-flight climb. In future studies, varying the length of this recovery period could help us explore whether 8-flight stair-climbing has creative thinking-enhancing effects within specific timeframes.

Our findings have several implications. Firstly, stair-climbing for 2 flights, which was completed in less than one minute, enhanced convergent thinking, a cognitive process evaluated over a span of four minutes. This underscores the potent efficacy of incorporating stair-climbing into daily routines as a means to invigorate convergent thinking. Secondly, our preliminary survey underscores the prevalent utilization of stairs for ascending 2 flights by the majority of individuals, which bolsters the pragmatic viability of advocating for the incorporation of two-flight stair-climbing in everyday scenarios. This revelation attests to the real-world applicability of our findings, offering a feasible option for integrating brief physical activity in routine life. Thirdly, although prevailing theories propound that physical activities of moderate to high intensities are more conducive to augmenting cognitive faculties (^46^; also referenced in^19^), our findings prompt a reconsideration of this paradigm. As delineated above, our results intimate that engagements in very light-intensity physical activities might be more efficacious for catalyzing enhancements in convergent thinking. This insight necessitates a thoughtful and strategic selection and alternation between varying types and intensities of physical activities in daily life, aligning them with the specific cognitive outcomes desired. Fourthly, our discovery that stair-climbing for 2 flights, considered a very light-intensity physical activity, led to an enhancement in convergent thinking and showed a trend toward improving originality, aligns with another strand of research demonstrating the cognitive benefits of non-aerobic activities. For instance, studies on mindful movements and yoga have indicated their positive effects on cognition, including divergent thinking^19,47,48^. These findings emphasize the significance of taking a comprehensive approach when assessing the relationship between exercise and cognitive effects. Such an approach transcends the notion of exercise as 'mindless' physical activity, offering a nuanced and informative framework for interpreting research outcomes. This perspective aligns with established viewpoints in both neuroscience and exercise-cognition research^49–52^.

## Conclusions

In conclusion, this study highlights the substantial impact of brief, very light-intensity physical activity, exemplified by two-flight stair-climbing, on augmenting convergent thinking, demonstrating its practical relevance and efficacy in daily life. Contrary to the prevalent emphasis on higher-intensity activities for cognitive benefits, our findings suggest that very light-intensity activities may also offer significant advantages, especially for convergent thinking, necessitating a strategic approach to selecting activity types and intensities to align with desired cognitive outcomes. The enhancements in convergent thinking were notably independent of mood alterations. Future research should further investigate the underpinning mechanisms and explore optimal activities for diverse cognitive improvements. Additionally, investigating the timeframes of cognitive improvements through variations in task durations and recovery periods, as well as exploring the benefits of qualitative aspects of exercise like mindful movements, represent promising directions for further investigation.

### Supplementary Information

Supplementary Information.

## Data Availability

The data that support the findings of this research are available from the corresponding author upon reasonable request.
