# Supplementary material for: Evaluating the impact of a short bout of stair-climbing on creative thinking in a between-subjects pretest posttest comparison study
Source: Sci Rep. 2024 Jan 2;14:176. doi: 10.1038/s41598-023-50282-2 (PMC10762161; doi:10.1038/s41598-023-50282-2)
Supplement: Supplementary file 1 — Supplementary Information. [file 41598_2023_50282_MOESM1_ESM.pdf]

Evaluating the impact of a short bout of stair-climbing on creative thinking in a between-subjects pretest posttest comparison study

Chihiro Kawashima, Chong Chen *, Kosuke Hagiwara, Tomohiro Mizumoto, Mino Watarai, Takaya Koga, Fumihiro Higuchi, Yuko Fujii, Emi Okabe, Shin Nakagawa

Supplementary material

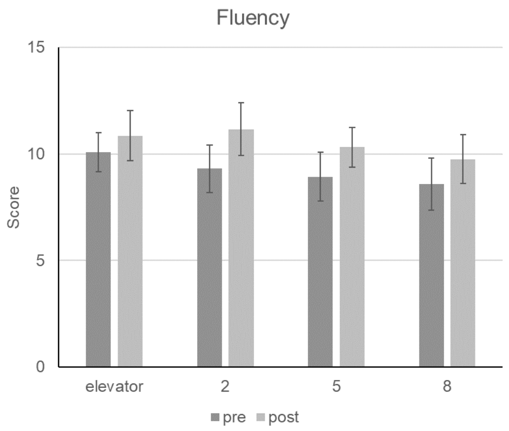

(a)

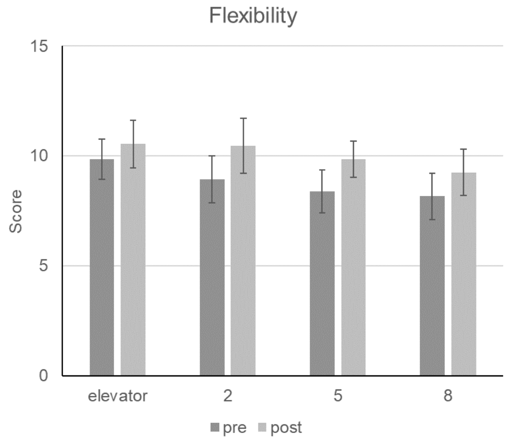

(b)

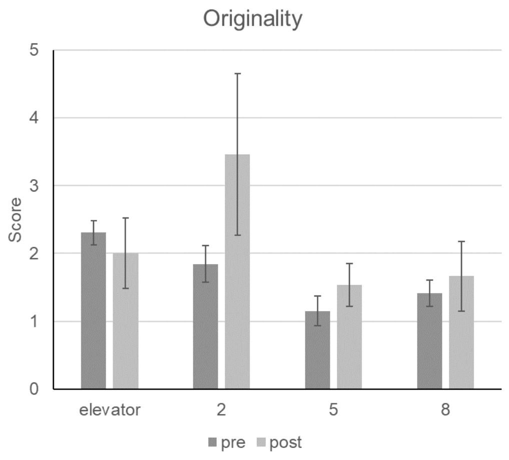

(c)

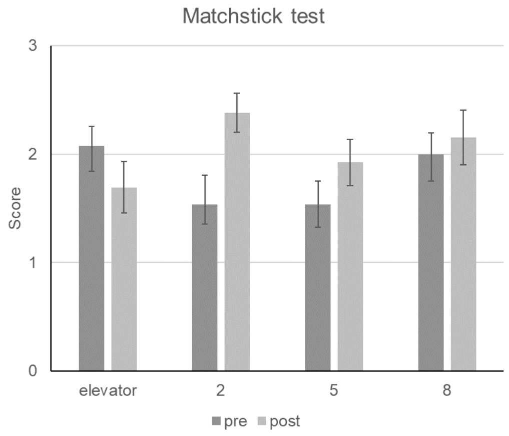

(d)

Figure S1. Scores of creative thinking pre- and post-intervention. (a)-(c): divergent thinking. (d): convergent thinking. Please see the manuscript for the results of time*intervention two-way repeated measures ANOVA. Notably, there was no pre-interventional differences in the scores of divergent and convergent thinking (all $p>0.16$, ANOVA).
